# Comparative Analysis of miRNA Expression Profiles of Yak Milk-Derived Exosomes at Different Altitudes

**DOI:** 10.3390/ani15010087

**Published:** 2025-01-02

**Authors:** Wenwen Ren, Yongfu La, Xiaoming Ma, Xiaoyun Wu, Xian Guo, Min Chu, Ping Yan, Xianyong Lan, Chunnian Liang

**Affiliations:** 1 Key Laboratory of Animal Genetics and Breeding on Tibetan Plateau, Ministry of Agriculture and Rural Affairs, Lanzhou Institute of Husbandry and Pharmaceutical Sciences, Chinese Academy of Agricultural Sciences, Lanzhou 730050, China; 2 Key Laboratory of Yak Breeding Engineering of Gansu Province, Lanzhou Institute of Husbandry and Pharmaceutical Sciences, Chinese Academy of Agricultural Sciences, Lanzhou 730050, China; 3 Key Laboratory of Animal Genetics, Breeding and Reproduction of Shaanxi Province, College of Animal Science and Technology, Northwest A&F University, Yangling 712100, China

**Keywords:** yak, milk-derived exosomes, miRNA

## Abstract

Yaks are a rare and unique animal species inhabiting the Qinghai–Tibet Plateau; they are renowned for their remarkable ability to thrive in harsh environments. Milk-derived exosomes, tiny vesicles containing various biological molecules, play crucial roles in numerous pathological and physiological processes, including cell growth, development, and immune regulation. This study delved into the microRNA expression profiles of yak milk-derived exosomes collected from both high- and low-altitude populations using small RNA sequencing. These miRNAs were found to be implicated in pathways associated with mammary gland inflammation, virus infection regulation, and heat stress response. Functional enrichment analyses, utilizing GO and KEGG databases, revealed that the target genes of these differentially expressed miRNAs are enriched in signaling pathways crucial for Th17 cell differentiation and the Ras-MAPK signaling pathway. In conclusion, this research illuminates the adaptive mechanisms of yaks through the differential expression of miRNAs in their milk-derived exosomes across varying environmental conditions. These findings provide a valuable foundation for future investigations into yak resilience and the potential of milk-derived exosomes as tools for disease management and immune modulation.

## Simple Summary

This study utilized small RNA sequencing to explore how the expression of miRNAs differs in milk-derived exosomes from yaks living at different altitudes. The analysis revealed distinct miRNAs associated with yak adaptations to environmental pressures such as mastitis, viral infections, and heat stress. These findings suggest that miRNAs carried by yak milk-derived exosomes play a crucial role in yak resilience, disease resistance, and immune regulation, offering valuable insights for a deeper understanding of yak adaptation.

## 1. Introduction

The yak (*Bos grunniens*) is a unique bovine species uniquely adapted to the challenging environment of the Qinghai–Tibet Plateau, where it thrives at altitudes exceeding 3000 m. Renowned as the “boat of the plateau” and “multi-purpose livestock” [1], yaks are highly abundant and well distributed across this high-altitude region. These hardy creatures demonstrate extraordinary resilience against extreme conditions: high altitudes (3000–6500 m), low oxygen pressure (below 10 mmHg), frigid temperatures (an average annual temperature below 0 °C), significant diurnal temperature variations (over 15 °C), short grazing seasons (100–120 days), and intense solar radiation. Compared to other mammals, yaks exhibit exceptional survival capabilities, displaying remarkable tolerance to hypoxia, cold, and radiation.

Exosomes are nano-sized extracellular vesicles (EVs) formed by a lipid bilayer membrane secreted by cells; these versatile vesicles range in size from 30 to 100 nanometers [1]. Milk-derived exosomes with the remarkable ability to circulate through bodily fluids and reach distant tissues stand out as potent delivery vehicles for transporting a diverse array of biomolecules and regulatory factors throughout the organism. Extensive research has illuminated the critical roles played by these biomolecules, carried by milk-derived exosomes, in numerous physiological and pathological processes, including cell growth, development, and immune regulation [2,3,4,5]. From a physiological standpoint, exosomes facilitate the intercellular transport of proteins and RNA. In immunology, they exhibit antigen-presenting capabilities [6].

MicroRNAs (miRNAs) are small, endogenous, non-coding RNAs, approximately 18–25 nucleotides in length. These tiny molecules wield significant regulatory power in both animals and plants by targeting mRNA for either cleavage or translational repression, and miRNAs are relatively conserved throughout biological evolution [7,8,9]. MiRNAs exert their regulatory function by imperfectly or perfectly binding to 3′-untranslated regions (3′-UTRs) of target mRNAs. This binding leads to either translational repression or mRNA degradation, thereby negatively regulating gene expression at the post-transcriptional level [10,11]. MiRNAs are a class of non-coding RNAs that play important roles in regulating gene expression. Although estimated to comprise only 2–5% of all mammalian genes, miRNAs are thought to regulate the expression of over 60% of protein-coding genes [12]. As a result, they are widely involved in regulating various organ morphogenesis and cellular activities, including proliferation, differentiation, regulation, and apoptosis [13].

The yak, a unique bovine species adapted to high-altitude environments, exhibits distinct physiological mechanisms and gene expression patterns compared to other cattle breeds. By collecting milk samples from yaks raised at different elevations, extracting exosomes, and performing high-throughput sequencing of miRNAs, this study aims to analyze the differential expression of these miRNAs across different altitudes. This research will contribute to a deeper understanding of how high-altitude environments influence miRNA regulation in yaks, providing molecular insights for explaining yak stress resistance. Furthermore, it will offer valuable guidance for optimizing yak husbandry practices and breeding strategies in high-altitude regions.

## 2. Materials and Methods

### 2.1. Collection of Milk

Milk samples were collected from six healthy yaks in lactation (4–5 years old). Three yaks were sourced from Xiahe County, Gannan Tibetan Autonomous Prefecture (altitude 3200 m, 102.567187° N, 34.827360° E), while the other three were from Angren County, Rikaze City, Tibet Autonomous Region (altitude 4587.5 m, 29.463654° N, 86.641097° E). Prior to milking, the udder area of each yak was thoroughly cleaned with warm water and then sanitized with a hot towel. Manual milking commenced, and the collected milk samples were immediately transferred to 50 mL sterile centrifuge tubes and immersed in dry ice. The samples were promptly transported to the laboratory and stored at −80 °C.

### 2.2. Exosomes Extraction and Identification

#### 2.2.1. Extraction of Exosomes by Ultracentrifugation

The samples were thawed at 37 °C. The thawed samples were transferred to new centrifuge tubes. Initially, samples were centrifuged at 2000× *g* at 4 °C for 30 min. The supernatant was carefully transferred to a new centrifuge tube and centrifuged at 10,000× *g* at 4 °C for 45 min to remove larger vesicles. The supernatant was collected and transferred to a new centrifuge tube. Ultracentrifugation was performed at 4 °C using an ultracentrifuge rotor at 100,000× *g* for 70 min. The supernatant was removed, and the pellet was resuspended in 1 mL of pre-chilled 1 × PBS (Sangon Bioengineering Co., Ltd., Shanghai, China) and stored at 4 °C.

#### 2.2.2. Purification of Exosomes Using Sucrose Cushion Method

First, a 1 mL volume of exosome sample was carefully layered onto 250 μL of a 30% sucrose cushion and centrifuged at 100,000× *g* for 70 min. Following centrifugation, 250 μL of the 30% sucrose cushion, located at the bottom, was carefully removed. This was diluted with PBS to 3 mL and centrifuged again at 100,000× *g* for 70 min, and the supernatant was then discarded. The precipitation was resuspended in 200 μL of pre-cooled PBS. An amount of 20 μL was used for electron microscopy imaging, 10 μL was used for nanoparticle size analysis, and the remaining exosomes were stored at −80 °C.

#### 2.2.3. Exosome Characterization Using Transmission Electron Microscopy

An amount of 10 μL of exosomes was added to the copper mesh for precipitation for 1 min, and the floating liquid was sucked off with filter paper. An amount of 10 μL of uranyl acetate (Xinrui Technology Co., Ltd., Shenzhen, China) was added dropwise to precipitate on the copper mesh for 1 min, and then the floating liquid was removed by filter paper again. After drying at room temperature for several minutes, electron microscope detection imaging was performed at 100 kv, and the transmission electron microscope imaging results were finally obtained.

#### 2.2.4. Exosome Particle Size Analysis

An amount of 10 μL of the exosomes was diluted to 30 μL. After the instrument performance test with the standard was qualified, the exosome sample could be loaded. Attention should be paid to the need for gradient dilution to prevent the sample from blocking the injection needle. The particle size and concentration information of exosomes detected by the instrument could be obtained after the sample was completed.

### 2.3. Total RNA Extraction of Exosomes

According to the manufacturer’s instructions, RNA was extracted from milk-derived exosomes using the Norgen 58,000 kit (Norgen Bio Inc., Thorold, ON, Canada).

### 2.4. Small RNA Sequencing

#### 2.4.1. Construction and Sequencing of Small RNA Library

The experimental workflow followed the standard procedures provided by Illumina, including library preparation and sequencing. The sRNA sequencing library was prepared using the TruSeq Small RNA Sample Prep Kit (Illumina, San Diego, CA, USA). After library construction, the prepared libraries were sequenced on an Illumina HiSeq 2000/2500 platform. Sequencing was performed with single-end reads of 1 × 50 bp in length (SE50).

#### 2.4.2. Data Quality Control

The original image data obtained by sequencing were subjected to base calling to obtain a sequence file stored in FASTQ format, called raw data or raw reads. The FASTQ file stores the sequence and base quality information of reads. Clean reads were obtained after the raw data were processed by quality control. The clean reads removed the 3′ linker and performed length screening to retain the sequence with a base length of 18–26 nt. The remaining sequences were compared with various RNA database sequences (excluding miRNAs), such as an mRNA database, RFam database (including rRNA, tRNA, snRNA, snoRNA, etc.), and Repbase database (repetitive sequence database), and filtered. The final data obtained are valid data and can be used for subsequent miRNA data analysis.

#### 2.4.3. Identification and Prediction of miRNA and Differential Expression Analysis

ACGT101-miR was used to identify miRNA and calculate the expression level of miRNA in each sample. The expression levels were statistically analyzed to evaluate the correlation of miRNA expression characteristics and differentially expressed miRNAs in samples within and between groups. When measuring the expression level, the norm value (normalized based on miRNA raw data) [14] was used as a measure of the miRNA expression level, and the expression level of miRNA in different samples was counted. The *p*-value calculation model based on normal distribution was used to calculate the *p*-value. The threshold value of $\left|\log_{2}(fold\;change\left.)\right|\geq 1\right.$ and *p* < 0.05 were used to screen differentially expressed miRNAs (DEMs).

#### 2.4.4. DEM Target Gene Prediction and GO and KEGG Enrichment Analyses

TargetScan (V5.0) and Miranda (v3.3a) software were used to predict the target genes of DEMs. The target genes predicted by TargetScan with a context score percentile below 50 were removed. For Miranda, predicted target genes with a maximum free energy greater than −10 were excluded. The final set of target genes for each differentially expressed miRNA was determined by taking the intersection of predictions from the two software. These predicted target genes were then subjected to GO (Gene Ontology) and KEGG (Kyoto Encyclopedia of Genes and Genomes) pathway enrichment analyses.

## 3. Results

### 3.1. Results of Exosome Extraction and Identification

**Figure 1:**
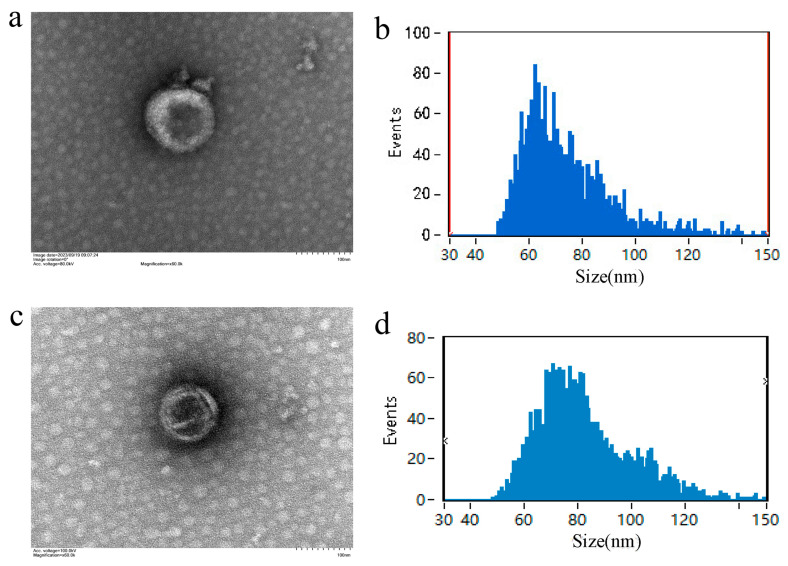
Exosome electron microscope and particle size. Note: (**a**) Electron microscopy of yak milk-derived exosomes at high altitude; (**b**) particle size distribution of yak milk-derived exosomes at high altitude; (**c**) electron microscopy of yak milk-derived exosomes at low altitude; (**d**) particle size distribution of yak milk-derived exosomes at low altitude.

Electron microscopy imaging revealed that exosomes isolated from yak milk, regardless of altitude, exhibited the typical morphology of spherical particles with an indented center (Figure 1). However, notable differences in particle size and concentration were observed between exosomes from high-altitude and low-altitude yaks. High-altitude exosomes displayed an average particle size of 73.50 nm and a concentration of 3.35 × 10^11^ particles/mL. In contrast, exosomes from low-altitude yaks had an average particle size of 81.95 nm and a concentration of 3.85 × 10^10^ particles/mL (Table 1).

### 3.2. Results of miRNA Sequencing of Milk-Derived Exosomes 

#### 3.2.1. An Analysis of the Characteristics of the Milk-Derived Exosomes’ miRNA

**Figure 2:**
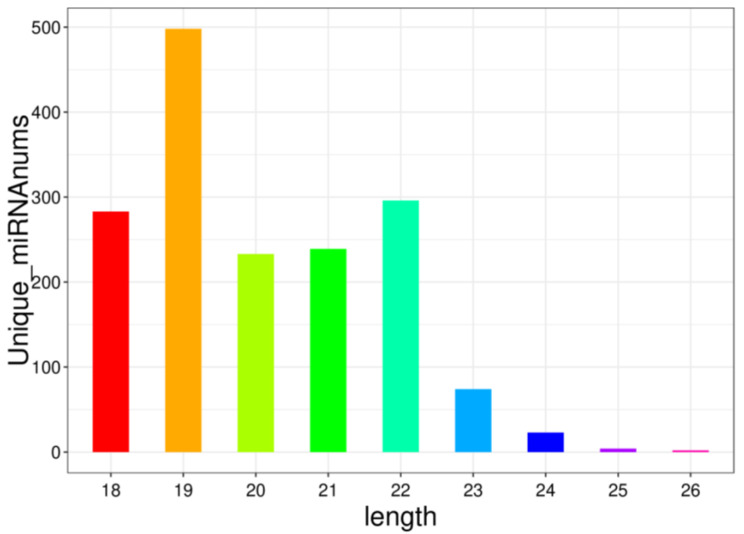
Length distribution of miRNA.

The analyzed data following quality control are presented in Table 2. Further miRNA identification and prediction analysis were conducted on the valid data. A statistical analysis of the identified miRNAs revealed a length distribution primarily within the 18–26 nt range, with the 19 nt length being the most prevalent. This observation aligns with the typical cutting pattern of the Dicer enzyme, further supporting the accuracy of the miRNA sequencing process (Figure 2).

**Figure 3:**
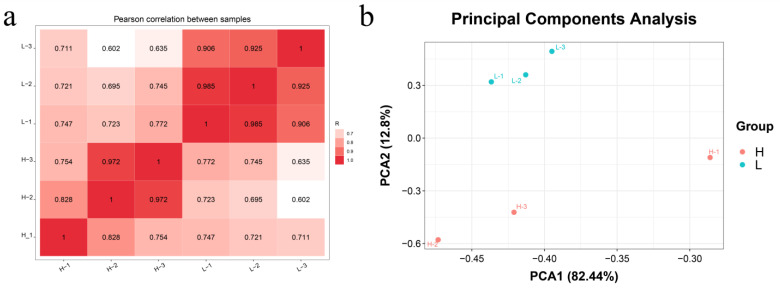
Correlation heat map and PCA clustering map between the two groups. Note: (**a**) correlation heat map; (**b**) PCA cluster diagram. H: yak milk-derived exosomes at high altitude; L: yak milk-derived exosomes at low altitude.

The results of the correlation heatmap demonstrate that intra-group correlations are higher than inter-group correlations (Figure 3a). Furthermore, a PCA reveals clear clustering of the samples within their respective groups (Figure 3b). These findings indicate good reproducibility within samples and support the reliability of the data.

#### 3.2.2. Differential Expression Analysis of DEMs

**Figure 4:**
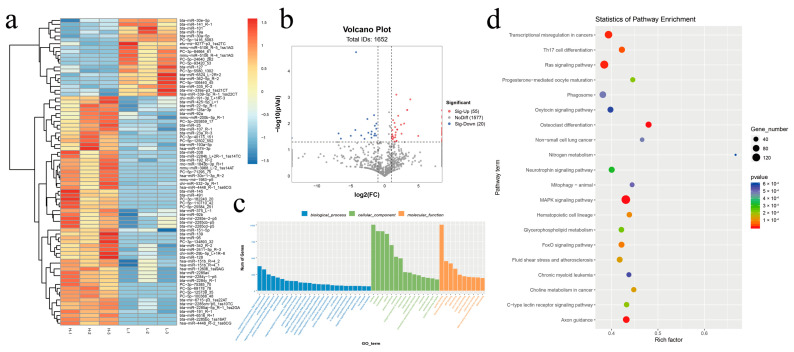
Differential expression analysis of miRNA. Note: (**a**) heat map of DEMs; (**b**) volcano plot of DEMs; (**c**) GO enrichment of DEM target genes; (**d**) KEGG pathway enrichment of DEM target genes.

A total of 75 DEMs were identified between the high-altitude and low-altitude yak milk-derived exosome samples, of which 55 were upregulated and 20 were downregulated (Figure 4a,b). To further investigate the biological functions regulated by these miRNAs, their target genes were predicted using both TargetScan and Miranda software. The intersection of these two lists served as the final target gene list, which was then subjected to GO and KEGG enrichment analyses. The analyses revealed significant enrichment in biological processes, such as the positive regulation of transcription in the DNA template, the positive regulation of transcription by RNA polymerase II, and protein phosphorylation. The enriched cellular components included the nucleus, cytosol, and nucleoplasm. The enriched molecular functions included protein binding, identical protein binding, and ATP binding (Figure 4c). Pathway analyses highlighted enrichment in the mitogen-activated protein kinases (MAPK) signaling pathway, the Ras signaling pathway, Th17 cell differentiation, and Axon guidance (Figure 4d).

## 4. Discussion

Milk, a valuable dietary component for both humans and infants, is renowned for its abundance of nutrients and bioactive molecules, including growth factors [15], metabolic hormones, and cytokines [16,17]. Milk is also known to contain a significant number of exosomes. Due to their unique bilayer membrane structure, exosomes show promise as drug carriers, offer potential for anti-inflammatory applications, and serve as valuable biomarkers for various health conditions [18]. This study extracted exosomes from yak milk using a density gradient centrifugation method. The extracted exosomes exhibited the typical appearance and particle size characteristics of milk-derived exosomes. Gao et al. [19] collected bovine milk samples from dairy farms in Beijing and yak milk samples from farms in Tibet and Gansu Province. They isolated milk-derived exosomes and performed miRNA sequencing, identifying 130 differentially expressed miRNAs, of which 51 were upregulated and 79 were downregulated in yak milk and milk-derived exosomes. Furthermore, they identified the top 20 miRNAs with relatively consistent high expression in yak milk-derived exosomes and found that bta-miR-34a acts as an effective regulator in mitigating hypoxia-induced damage to IEC-6 cells. Studies have shown that HIF-α expression is upregulated in the inflamed mucosa of patients with inflammatory bowel disease and regulated by various inflammatory stimuli. Compared with cow milk-derived exosomes, yak milk-derived exosomes enhance PHD-1 expression and suppress HIF-α and its downstream target VEGF expression under hypoxia. These exosomes exhibit a more pronounced effect on promoting the growth of intestinal epithelial cells (IECs), increasing IEC proliferation, and contributing to strengthening the intestinal epithelial barrier function. Furthermore, yak milk-derived exosomes more effectively activate the hypoxia-inducible factor signaling pathway [20].

This study identified 75 differentially expressed miRNAs, of which 55 were upregulated and 20 were downregulated, and several key miRNAs were identified. Several key miRNAs, including bta-miR-30a-5p and members of the bta-miR-2285 family, emerged as critical regulators in inflammatory processes. The bta-miR-30a-5p miRNAdirectly targets TRAM, a protein tightly associated with the NF-κB signaling pathway. Notably, transfection experiments confirmed that bta-miR-30a-5p suppresses the activation of the LPS-induced NF-κB pathway and the expression of downstream pro-inflammatory factors. The expression of its potential target gene, TRAM, was also inhibited [21]. Furthermore, bta-miR-30a-5p plays a crucial role in regulating the infection process of the bovine mammary epithelial cells (BMECs) triggered by LPS [22]. The bta-miR-2285 family consists of over 40 members, spanning the entire bovine genome [23]. Among the 75 differentially expressed miRNAs identified, 7 belonged to the bta-miR-2285 family. Specifically, bta-miR-2285aj-5p was found to be significantly upregulated in BMECs stimulated with lipoteichoic acid. Bta-miR-2285aj-5p appears to induce mastitis by activating the ERK1/2 and NF-κB pathways, leading to the positive regulation of SPDYA and FGL1 [24]. Bovine mastitis-related long non-coding RNA acts as a molecular sponge for bta-miR-145. CBFB is one of bta-miR-145′s 23 target genes. The bta-miR-145/CBFB pathway plays a role in Staphylococcus aureus-induced mastitis by influencing inflammatory responses and self-protective mechanisms [25]. Additionally, bta-miR-145 exhibited significant differential expression between mammary tissues of Holstein cows under heat stress compared to normal conditions. This miRNA appears to function as a key regulator during heat stress in Holstein cows, mitigating heat stress damage [26].

Several miRNAs have been shown to play roles in bovine viral and parasitic infections. Bta-miR-30e-5p, bta-miR-339a, and bta-miR-92a show altered expression patterns in the serum of calves infected with bovine viral diarrhea virus (BVDV) over time [27]. Similarly, bta-miR-2411 is significantly upregulated by over 2.1-fold in Madin-Darby bovine kidney (MDBK) cells 8 h after infection with BVDV. The overexpression of bta-miR-2411 leads to significant reductions in both BVDV mRNA levels and viral titers [28]. Following infection with bovine ephemeral fever virus (BEFV), bta-miR-101 is significantly upregulated in MDBK cells. Acting as an antiviral host factor, bta-miR-101 inhibits viral replication by targeting the NF-κB-repressing factor and suppresses BEFV-induced apoptosis [29]. Bta-miR-141 and bta-miR-191 have emerged as potential antimicrobial targets within bovine alveolar macrophages [30]. The expression level of bta-miR-23a gradually increases over time in response to infection with the *fasciola gigantica* [31].

Heat stress significantly impacts dairy cows, and several miRNAs have been identified as potential biomarkers and regulators of this response. Studies have shown differential expression of miRNAs, including bta-miR-30a-5p and bta-miR-19a, and members of the bta-miR-2284 family exhibiting differential expression in both pregnant and non-pregnant dairy cows. This differential expression suggests their involvement in the physiological and immune responses triggered by heat stress [32]. Further research suggests that the target genes of the bta-miR-2284 family are likely involved in heat-induced immune responses, although their precise biological roles remain unknown [33]. Bta-miR-25 may influence the MAPK pathway, potentially through the c-Jun N-terminal kinase (JNK) gene, thereby impacting mammary gland function in heat-stressed dairy cows [34]. Beyond heat stress, certain miRNAs exhibit distinct expression patterns during pregnancy. Specifically, bta-miR-128 and bta-miR-127 can be used as specific exosome miRNAs in early pregnancy (60d) and middle pregnancy (150d) [35]. Additionally, bta-miR-127 expression changes in BMECs after cadmium exposure. This suggests a potential role for this miRNA in regulating apoptosis and immune responses within these cells [36].

This study uncovered a significant enrichment of target genes for differentially expressed miRNAs in functions and signaling pathways related to protein binding, the MAPK signaling pathway, and Th17 cell differentiation. Th17 cells are a subset of helper T cells that play a crucial role in the pathogenesis of many autoimmune diseases [37]. MiRNAs can act as both positive and negative regulators of Th17 differentiation, influencing the development of these cells with either inductive or suppressive effects [38]. For instance, miR-326 targets ETS1, a negative regulator of TH17 cell differentiation, and promotes TH17 cell differentiation in vitro [39]. Conversely, the increased expression of miR-10a in CD4(+) T cells has been observed to suppress Th17 cell differentiation [40]. MAPKs signal transduction has emerged as a potential regulatory mechanism for T lymphocyte development and effector response. Growing evidence suggests that MAP3K1 plays a role in Th17 cell signal regulation and IL-17 expression [41]. MEKK1, encoded by MAP3K1, also modulates Th17 differentiation by regulating cell cycle inhibitory genes like Cdkn1b. The loss of MAP3K1 in T cells results in increased IL-17 production and differentiation into Th17 [42]. Furthermore, numerous studies have highlighted the importance of miRNAs targeting the RAS-MAPK pathway in cancer development. The RAS/MAPK axis regulates key cancer cell behaviors, including proliferation, apoptosis, inflammation, migration, and metastasis. Tumor suppressor miRNAs can interact with oncogenic KRAS, thereby inhibiting RAS/MAPK signaling [43,44].

## 5. Conclusions

This study extracted exosomes from the milk of high- and low-altitude yaks and performed small RNA sequencing. The results reveal that differentially expressed miRNAs may regulate yak mastitis, viral infection responses, and thermotolerance by influencing Th17 cell differentiation and the RAS-MAPK signaling pathway. Overall, this research expands the understanding of yak miRNAs and provides valuable reference information for future investigations into their roles in yak biology. Further research will focus on the selected candidate miRNAs and explore their expression patterns in yak mammary epithelial cells and immune cells. Moreover, in vitro experimental models will be used to investigate the effects of these candidate miRNAs on yak resistance to mastitis, responses to viral infections (using relevant viral models), and tolerance to heat stress, aiming to elucidate their potential roles in yak immune function and stress response.

## Figures and Tables

**Table 1:** The particle sizes and concentrations of yak milk-derived exosomes at different altitudes.

Sample	Particle Size (nm) (Means ± SD)	Concentration (Particles/mL)
H	73.50 ± 16.20	3.35 × 10^11^
L	81.95 ± 16.79	3.85 × 10^10^

Note: H: yak milk-derived exosomes at high altitude; L: yak milk-derived exosomes at low altitude.

**Table 2:** Raw reads and valid reads in yak milk-derived exosomes at high and low altitudes.

Samples	Raw Reads	3ADT&Length Filter	Junk Reads	Clean Reads	Rfam	mRNA	Repeats	Valid Reads
H_1	4,486,944	3,029,790	19,258	3,010,532	68,347	27,015	2015	1,348,282
H_2	3,294,479	1,699,628	32,365	1,667,263	59,885	34,108	2395	1,474,858
H_3	3,296,214	1,981,630	18,735	1,962,895	61,322	32,951	2421	1,207,344
L_1	2,537,372	1,773,612	8420	1,765,192	25,033	10,208	504	722,013
L_2	2,418,352	1,855,403	6871	1,848,532	18,948	12,152	677	527,095
L_3	2,239,586	1,742,244	7334	1,734,910	18,133	11,202	764	462,697

## Data Availability

The data presented in this study are openly available at Gene Expression Omnibus (GEO), reference number GSE282381.

## Abbreviations

BEFV	bovine ephemeral fever virus
BMECs	bovine mammary epithelial cells
BVDV	bovine viral diarrhea virus
DEMs	differentially expressed miRNAs
EVs	extracellular vesicles
IEC	intestinal epithelial cells
MAPKs	mitogen-activated protein kinases
MDBK	Madin-Darby bovine kidney
miRNAs	microRNAs
3′-UTRs	3′-untranslated regions
